# Jaffe-Campanacci syndrome or neurofibromatosis type 1: a case report of phenotypic overlap with detection of NF1 gene mutation in non-ossifying fibroma

**DOI:** 10.1186/s13052-020-0813-9

**Published:** 2020-05-11

**Authors:** Silvia Vannelli, Raffaele Buganza, Federica Runfola, Ilaria Mussinatto, Antonio Andreacchio, Luisa de Sanctis

**Affiliations:** 1 Pediatric Endocrinology Unit, Department of Public Health and Pediatric Sciences, Regina Margherita Children’s Hospital, University of Turin, Turin, Italy; 2 Department of Public Health and Pediatric Sciences, Postgraduate School of Pediatrics, Regina Margherita Children’s Hospital, University of Turin, Turin, Italy CAP 10126; 3 Department of Pediatric Orthopedic Surgery, Regina Margherita Children’s Hospital, University of Turin, Turin, Italy

**Keywords:** Neurofibromatosis type 1, Jaffe-Campanacci syndrome, Non-ossifying fibromas, Giant cell granulomas of the jaw, café-au-lait macules

## Abstract

**Background:**

Jaffe-Campanacci syndrome is characterized by multiple non-ossifying fibromas, café-au-lait macules and giant cell granulomas of the jaw. Even if the association between all these peculiar features and neurofibromatosis type 1 have been described, it has not yet been clarified whether Jaffe-Campanacci syndrome represents a distinct entity or it can be regarded as a neurofibromatosis type 1 subtype.

**Case presentation:**

The patient here described is a young boy, who fulfilled the clinical diagnostic criteria for both syndromes. He had a complex clinical history with café-au-lait macules, axillary and inguinal freckling, multiple non-ossifying fibromas, giant-cell granuloma of the jaw, neurofibromas, plexiform fibroma, ocular Lisch nodules, optic chiasmatic- hypothalamic glioma, pseudarthrosis, scoliosis, short stature, vascular anomalies, seizures. Molecular analysis of the NF1 gene both on blood cells and non-ossifying fibroma’s biopsy tissue allowed the detection of a novel variant within the coding region, NM_000267.3:c.2789_2791delATC(p.Tyr930_Pro931delinsSer), with loss of heterozygosity (second hit mutation) in the non-ossifying fibroma.

**Conclusion:**

This result indicates that every patient with clinical features of Jaffe-Campanacci syndrome should be further evaluated to detect features related to neurofibromatosis type 1 and genetically investigated for mutations in the NF1 gene, since this could lead to a definite diagnosis, but also could clarify and quantify the real genotype-phenotype overlap between neurofibromatosis type 1 and Jaffe-Campanacci syndrome.

## Background

The eponymous Jaffe–Campanacci syndrome (JCS) was coined in 1982 by Mirra [1] on the basis of a set of signs and symptoms firstly described by Jaffe [2] and Campanacci [3], encompassing café-au-lait macules (CALMs), multiple non-ossifying fibromas (NOFs) and central giant cell granulomas (CGCG) of the jaw [1, 2, 4]. Other features variably included in the JCS are mental retardation, hypogonadism, cryptorchidism, several congenital ocular anomalies and cardiovascular malformations (i.e. mitral insufficiency and stenosis of the aortic isthmus) [3]. Single reports have further described precocious puberty, alopecia, kyphoscoliosis, mega-ureter, chylothorax and chylopericardium [3, 4] within JCS.

Since its first description by Jaffe, it has been debated whether this syndrome represents a distinct entity or is a neurofibromatosis type 1 (NF1) subtype. The clinical diagnosis of NF1 is based on the presence of six or more CALMs > 5 mm in prepubertal and > 15 mm in postpuberal individuals, two or more neurofibromas of any type or one plexiform neurofibroma, freckling in the axillary or inguinal regions, optic glioma, two or more Lisch nodules, distinctive osseous lesion (such as sphenoid dysplasia or tibial pseudarthrosis), a first-degree relative harboring NF1 gene mutation [5].

Indeed, the three main signs of JCS (CALMs, NOFs and CGCG) have also been reported in NF1 patients and JCS case reports often lacked NF1 clinical or genetic exclusion [6].

## Case presentation

The first clinical manifestation in our patient was the appearance of CALMs at 1 month of age, afterwards increased in number until 20 macules with a diameter greater than 1.5 cm. Axillary and inguinal freckling, widespread neurofibromas (cutaneous and maculopapular in the trunk and limbs, subcutaneous and nodular in the head), an axillary plexiform neurofibroma and ocular Lisch nodules were subsequently detected, leading to the clinical diagnosis of NF1. In the second year of life a voluminous optic chiasmatic-hypothalamic glioma was found; surgery was excluded in consideration of the localization and large extension of the mass that was successfully treated with chemotherapy. At 9 years of age a spontaneous fracture occurred at lower limbs; X-rays revealed bilateral multiple lytic areas in the distal part of femur, distal and proximal part of tibia and fibula. Bone analysis on a sample taken during the surgery led to the diagnosis of NOFs. NF1 gene analysis both on blood cells and NOF tissue allowed to detect a novel variant: NM_000267.3:c.2789_2791delATC(p.Tyr930_Pro931delinsSer), “likely pathogenic” according to ACMG-AMP guidelines (criteria PM1+ PM2+ PM4+ PP3+ PP4) [7] with also loss of heterozygosity in the NOF. The parents did not show signs of NF1 or JCS but they decided not to undergo molecular analysis. Family history was unremarkable.

**Fig. 1:**
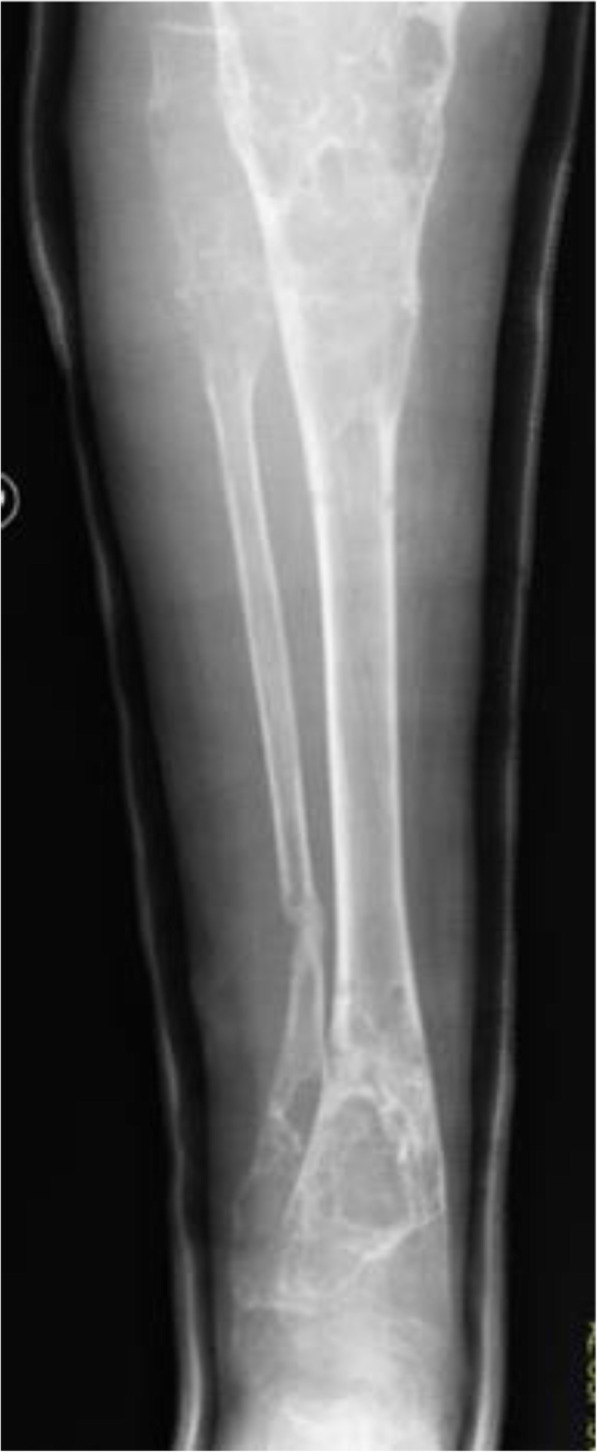
NOFs in the right leg

The monitoring of NOFs showed that they evolved into spontaneous fractures in the right femur, right humerus, right tibia, left femur (Fig. 1); furthermore, over time pseudarthrosis on both tibias, mild scoliosis and valgus deviation of the left knee have developed.

**Fig. 2:**
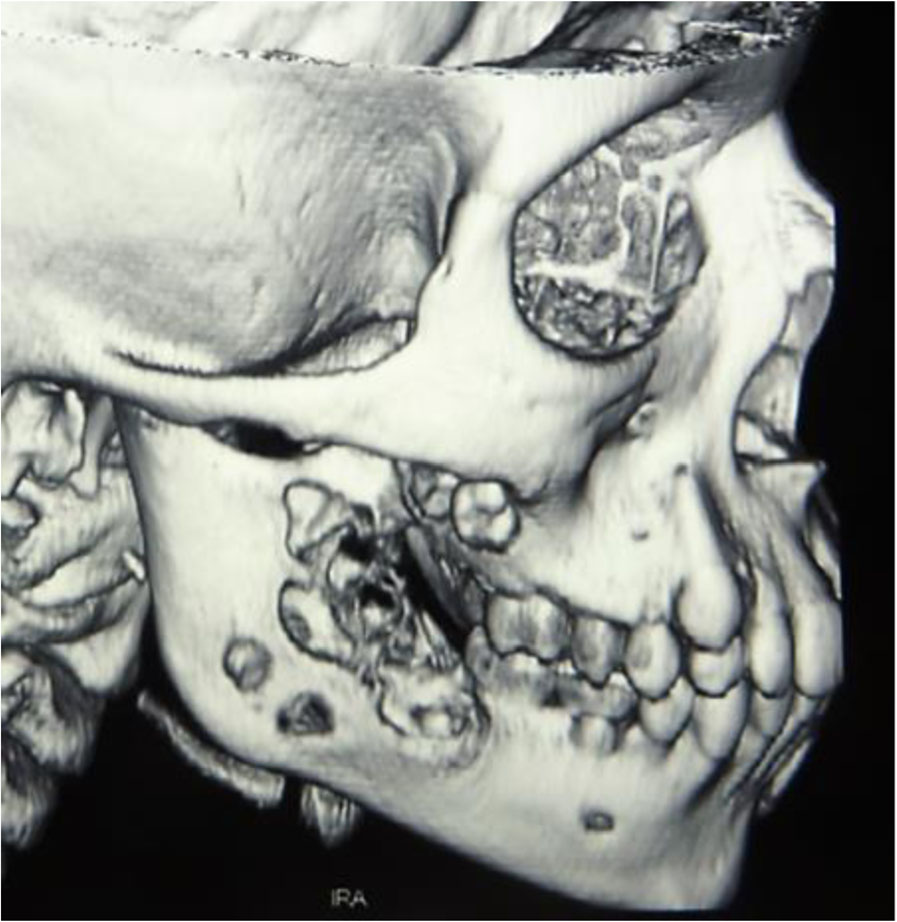
Giant-cell granuloma of the jaw

At the age of 11, a lesion appeared on the oral cavity floor, CGCG of the jaw was diagnosed from a bioptic specimen (Fig. 2) and then surgically removed.

The boy had short stature; the height was between the 50° and the 75° percentile according to the standards of Tanner [8] up to 9 years of age, then the growth rate decreased due to growth hormone deficiency, likely related to the previous optic chiasmatic- hypothalamic glioma. At 16.7 years of age the height was 162.5 cm, just above the 3rd percentile, not reaching the target height. Additional features displayed by our patients were hypertension, due to stenosis of the renal arteries and treated with two stenting procedures, abdominal aorta stenosis with coarctation treated with aortic angioplasty, diffuse unidentified bright objects (UBOs) in cerebral magnetic resonance and seizures (at 5 years of age with left temporoparietal electroencephalographic abnormalities, treated with oxcarbazepine for 4 years). At the moment, the boy is 17 years old and is on a multidisciplinary follow up.

## Discussion

By describing the new clinical entity for the first time, Jaffe suggested that the disorder was an unusual form of neurofibromatosis [2]. JCS reports have then been presented for years as distinct forms from NF1 [4, 9–15]. In the 2013 “WHO Classification of tumours of soft tissue and bone”, JCS is therefore defined as the association of NOFs and NF1 [16], but NOFs, considered for years as a distinctive feature of JCS, have also been reported in NF1 patients [17–29].

Fibrous cortical defects and NOFs are indeed common bone focal lesions, with an estimated rate up to 30% of the asymptomatic population in the first and second decade of life [30]. They are usually incidentally found in X-rays performed for injuries and belong to the group of developmental abnormalities [30]. In a series of 900 patients with biopsy-proven NOFs, Moser et al. found 72 cases (8%) with multiple lesions and the incidence of neurofibromatosis in presence of multiple NOFs was 5% [18]. Mankin et al. reported 401 patients with bone lesions described as NOFs and fibrous cortical defects, but only two of them meeting the criteria for JCS [31].

NOFs in JCS cases are mainly large and located in the metaphyseal region, with high number of anatomic sites involved and multiple small fractures, irregular ossification and cortical irregularity [31]. The most common sites at which NOFs occur are distal femur, proximal and distal tibia, proximal humerus, fibula and radius, with frequent fractures and some deformities of the involved bones [31]. In JCS the fracture risk appears to be high, as more than half of the patients will experience at least one fracture; it may be related to the lesion features, which are multiple and large, with thinning of the cortex and weakening of the weight-bearing bones [9]. The histology of NOFs is characterized by fibroblastic, often highly cellular, collagenous stromal tissue with few multicentric giant cells along with foci of xanthomatous reaction and few foamy histiocytes; sites of necrosis may be observed [31].

Baumhoer et al. found heterozygous NF1 gene mutation in two NOFs sample in patients with NF1.

[32]. Colby et al. described 4 patients with NOFs, who met the criteria of both JCS and NF1; the molecular test of the NF1 gene was performed in one patient (only in the blood), showing partial deletion of the gene [17]; the author thus suggested a genetic evaluation in all patients diagnosed with JCS and a radiographic screening of both knees to detect NOFs in NF1 during early adolescence or adulthood [17]. However, ionizing radiations should be used with caution in NF1 patients, especially in paediatric population and therefore clinicians should keep in mind the association between NF1 and NOFs and definitely consider radiographic examination in case of suggestive symptoms.

The other key feature of JCS, presented by our patient, is CGCG involving the jaw. In the Stewart’s series, somatic second-hit mutations in the NF1 gene were detected in two giant cell lesions from two unrelated patients with the main features of JCS [33]. CGCG has also been described in NF1 patients [34–43].

Some authors have hypothesized that the CGCG represented the mandibular manifestation of the long bone NOF, but Slootweg, performing a histologic comparison of a significant number of lesions, concluded that, although they may exhibit histologically similar areas, the two entities are separate [44]. He evidenced important differences: the fibrous stroma with the cells arranged in whorled bundles, which was the hallmark of NOFs, was observed in some, but not all CGCG (and in CGCG it was only focally); foam cells were found in almost a quarter of the NOFs but almost universally absent in CGCG; bone formation played a major part in CGCG whereas in NOFs it was nearly absent [44].

The third main feature of JCS is CALM, which is also one of the main signs of NF1. The pathophysiology is still largely unknown. De Schepper et al. reported that in NF1 patients, between normal skin and CALMs, higher density of melanocytes was present in CALMs; CALMs in control and NF1 patients differed in melanocyte density, melanin content and melanogenesis of melanocytes and in the amount of stem cell factor secreted by fibroblasts, which are all higher in NF1 [45].

JCS in not the only syndrome showing phenotypic overlap with NF1: other examples are represented by the ‘neuro-cardio-facial-cutaneous’ syndrome (with a variable degree of cognitive impairment, facial dysmorphism, congenital heart defects and skin abnormalities), related to mutations in the genes that encode other components of the RAS-MAPK pathway, such as PTPN11, KRAS8 and SOS1 in Noonan syndrome, PTPN11 in LEOPARD syndrome, HRAS13 in Costello syndrome, KRAS14, BRAF14,15, MEK1 and MEK2 in cardio-facio-cutaneous syndrome [46]. Another noteworthy example is Legius syndrome, due to SPRED1 mutations (RAS-MAPK pathway), whose phenotype includes café-au-lait spots, freckling and lacks the non-pigmentary manifestations of NF1; additional clinical manifestations reported commonly include lipomas, macrocephaly, learning disabilities, attention deficit, hyperactivity disorder, developmental delays, Noonan syndrome-like facies and, less frequently, short stature, pectus anomalies, depigmented macules, vascular lesions, headaches, hearing loss, seizures, polydactyly, scoliosis.

[46, 47]. Different signs of this syndrome are present also in our patient, so it is evident that overlapping phenotype can complicate the diagnosis.

The patient reported in our case fulfilled NF1 diagnostic criteria. He displayed café au lait macules, neurofibromas, plexiform neurofibroma, freckling in the axillary and inguinal regions, optic chiasmatic- hypothalamic glioma, Lisch nodules, osseous lesion such as tibial pseudarthrosis; he then developed scoliosis, UBOs, seizures, short stature, hypertension due to renovascular diseases, mid-aortic syndrome. All these features had previously been described in patients with NF1 [5], but it is very difficult to find them all in a single subject, which also showed also the JCS main signs in a severe form with a high number of NOFs. Here we reported an extremely severe case, which required a multidisciplinary approach with molecular analysis. A mutation on the NF1 gene, not yet reported in NF1, was detected either in blood cells and NOF’s tissue, pointing out the NF1 gene alterations can be responsible for all the clinical features, ascribed either to the JCS or NF1 conditions. Pathogenic germline NF1 mutations were identified by Stewart et al. in 13 out 14 patients with multiple café-au-lait macules and multiple NOFs or giant cell lesions (none of the mutations were detected directly on NOF tissue) [33]. All 13 subjects also fulfilled the diagnostic criteria for NF1. Consequently, Stewart et al. suggested that many JCS cases may actually have NF1 and they proposed a clinical algorithm for patients with NOFs and/or giant cell lesions [33]. They also recommend that the term “Jaffe–Campanacci syndrome” should only be used for patients with multiple CALMs, multiple NOFs, and/or CGCG who also lack a germline NF1 mutation or who lack evidence of an other recognized disorder associated with NOFs and CGCG [33].

The true incidence of NF1 mutations in JCS patients still remains uncertain, since molecular studies on the NF1 gene have not been so far performed in most JCS patients.

The cellular and molecular pathophysiology of the different features of the NF1 disease is not completely understood. Factors that complicate his understanding include the large size of the NF1 gene, the presence of several NF1 pseudogenes, the complex interactions between cell types, and the NF1-haploinsufficient state of all cells in the body [48]. For some features, a somatic inactivation of the wild type NF1 allele was detected, according to the “two- hit theory”: the first mutations in the NF1 gene could occur during embryonic development, generating NF1 mosaic patients and therefore another somatic NF1 mutations could represent the “second hit”, which inactivates the wild type NF1 allele in NF1 heterozygous patients [49]. Maertens et al. demonstrated the biallelic inactivation of the NF1 gene in melanocytes in NF1-related CALMs and evidenced that the mosaic phenotype reflected the embryonic timing and, accordingly, the neural crest–derived cells in the somatic NF1 mutation [48]. Other authors have described the same “second hit” mechanism with biallelic inactivation on NF1 gene in other tissues and related tumors [33, 38, 49, 50], including giant cell granulomas of the jaw in patients with NF1 [38] and JCS [33]. In our case, the molecular analysis of NF1 gene in the NOF detected NF1 mutation and loss of heterozygosity (only the mutant allele was present), which should be considered as the “second hit”.

In NF1, given the paucity of NF1 genotype–phenotype correlations, it has been proposed that, besides a phenotypic heterogeneity among the different variants within the gene, a role of modifier genes could explain the variability of clinical manifestations [50]. Many questions remain about the relevance of possible interaction partners and the function of neurofibromin protein domains in NF1 mutation [50]. All these processes may explain the different presentation of NF1 and maybe the characteristics of syndromes with phenotypic overlap, as JCS. Therefore, in these conditions genetic analysis can lead to the correct diagnosis, thus allowing also appropriate follow-up protocols as well.

## Conclusions

Here is described a patient displaying clinical features of both NF1 and JCS and a NF1 gene mutation detected on blood cells and NOF’s biopsy tissue, with loss of heterozygosity in the NOF. These findings support the thesis that JCS could be considered a NF1 subtype. Clinicians must always consider the possible presence of NOFs and CGCG in patients with NF1, paying attention to specific symptoms. On the other hand, in patients with signs of JCS, deeply evaluation is crucial to detect other features related to NF1. In consideration of the clinical and molecular overlapping characteristics between JCS and NF1, we strongly recommend to investigate for NF1 gene mutations in patients diagnosed as having the JCS.

## Data Availability

Data sharing not applicable to this article as no datasets were generated or analyzed during the current study.

## Abbreviations

JCS: Jaffe-Campanacci syndrome
NF1: Neurofibromatosis type 1
NOFs: Non-ossifying fibromas
CGCG: Central giant cell granulomas
CALMs: Café-au-lait macules
